# Covid-19 and non-communicable diseases (NCDs) in Africa: a narrative review

**DOI:** 10.4314/ahs.v23i3.48

**Published:** 2023-09

**Authors:** Yusuff Azeez Olanrewaju, Amos Abimbola Oladunni, Kenneth Bitrus David, Yusuf Olalekan Babatunde, Ibrahim Abdulmumin Damilola, Oluwakorede Adedeji, Colette Chidozie Ahamefula

**Affiliations:** 1 Faculty of Pharmacy, University of Ibadan, Oyo, Nigeria; 2 Faculty of Pharmaceutical Sciences, Ahmadu Bello University, Zaria, Nigeria; 3 Hull York Medical School, University of Hull, Hull, United Kingdom; 4 Faculty of Pharmaceutical Sciences, Kaduna State University, Kaduna, Nigeria; 5 Faculty of Pharmaceutical Sciences, University of Ilorin, Ilorin, Nigeria; 6 Faculty of Pharmacy, Delta State University, Abraka

**Keywords:** COVID-19, Pandemics, Africa, Non-Communicable Diseases

## Abstract

Coronavirus disease 2019 popularly known as COVID-19 is the current pandemic ravaging the world. It has disrupted so many aspects of humans' life including the healthcare systems of all countries. While governments have instituted preventive measures such as social distancing, self-isolation and lockdown in a bid to control the spread of the virus, the absence of vaccine can lead to poor management of key risk factors (including unhealthy diets and physical inactivity) associated with NCDs and limited access to preventive health services can further contribute to development and progression of NCDs. This study provides a review of available evidences from PubMed, google scholar, online databases, and papers from other sources on the impact of COVID-19 pandemic on NCDs in Africa and emphasizes lessons from past pandemics that can be adopted to reduce the burden of the disease.

## Introduction

Africa has a land mass of approximately 30 million square kilometers, with a cumulative population of approximately 965 million people; which constitutes about 14% of world population^1^. The COVID-19 pandemic poses as one of the greatest challenges to public health of the last century. SARS-CoV-2 is a virulent and contagious virus which has spread immensely round the globe, causing severe illness and widespread social and economic disruptions^2^,^3^. COVID-19 has scourged the world resulting in its declaration by the World Health Organization (WHO) as a pandemic on the 11th March, 2020 and it has become one of the deadliest pandemics in the last century 4,5. COVID-19 has affected more than 207,000 people in at least a hundred and sixty-six countries as of March 19, 2020 4. The absolute number of deaths have surpassed 8500 on a global scale and this value is expected to increase because the disease spreads rapidly 4.

Non communicable diseases (NCDs) kill 41 million people every year accounting for 71% of all global deaths with 80% of these deaths occurring in low- and middle-income countries (LMIC) where many health care services are limited^5^-^10^. LMIC are more vulnerable to NCDs as they have the least capacity to cope with the increasing burden due to poverty and according to the World Bank, eighty percent of the 54 African countries are classified as LMIC^10^-^11^.

The disease continues to ravage health and economic metrics globally, including progress on the NCDs global action plan. The pandemic has had widespread health impacts^12^, revealing the particular vulnerability of those with underlying conditions. Although the mortality rate of COVID-19 is quite low^11^, children and adults with existing comorbidities, most particularly NCDs such as hypertension, obesity and diabetes continue to be the most vulnerable group^13^. While governments have instituted preventive measures such as social distancing, self-isolation and lockdown in a bid to control the spread of the virus, the absence of vaccine can lead to poor management of key risk factors (including unhealthy diets and physical inactivity) associated with NCDs and limited access to preventive health services can further contribute to development and progression of NCDs. This study provides a review of available evidences on the impact of COVID-19 pandemic on NCDs in Africa and emphasizes lessons from past pandemics that can be adopted to reduce the burden of the disease. This study therefore seeks to answer the question: what is the extent of COVID-19 on global NCDs target in Africa.

## Method

We conducted a narrative review of published articles on COVID-19 and non-communicable diseases. PubMed and Google Scholar were searched with the following keywords: Covid-19, Coronavirus disease, non-communicable diseases, past pandemics, non-communicable disease target, COVID-19 prevention strategies, and non-communicable diseases burden in Africa.

## Result

### Burden of NCDs in Africa

According to the WHO, Non-Communicable Disease (NCD) refers to any medical condition or disease that is non-infectious and cannot be transmitted from one person to another. For example, Parkinson's disease, autoimmune disease, stroke, cardiovascular disease, osteoarthritis, osteoporosis, Alzheimer's disease, cataracts, respiratory disease, cancer etc.^14^,^15^. The Major NCDs in Africa ae cardiovascular diseases, cancers, chronic respiratory disease and diabetes^16^ with common behavioural risk factors including; tobacco use, harmful alcohol use, physical inactivity, and unhealthy diet^17^, ^8^. Between 1990 and 2015, deaths related to NCDs in Sub-Saharan Africa increased from 1.7million (25%) to 2.7million (34%) with cardiovascular diseases being the leading cause of deaths 19. Deaths from NCDs are increasing in Africa faster than anywhere else in the world and this is slowing economic development, undermining the attainment of sustainable development goals (SDGs) and increasing social inequality 20. In the ranking of the leading causes of Disability adjusted life years -DALYs (years of healthy life lost to premature death and disability), cardiovascular diseases ranked 6th, with diabetes, mental and substance use disorders and cancer ranking 9th, 10th, and 11th position 21. Cardiovascular diseases caused nearly 1million deaths in 2013 in Sub-Saharan Africa constituting 38.3% of non-communicable disease deaths^22^ and in the same year, Cancer caused 12% of NCDs deaths with large variations across regions in sub-Sahara Africa^20^. Chronic respiratory diseases and diabetes has caused 10% and 5% of total deaths in Sub-Saharan Africa^23^. NCD risk factors such as High blood pressure, poor diet, air pollution, high body-mass index, tobacco smoking, alcohol and drug use, high fasting plasma glucose, high total cholesterol, low physical activity are among the top 10 global risk factors for death^23^. It has been estimated by WHO that deaths from NCDs are likely to increase by 17% over the next 10years and that Africa will experience a 27% increase i.e., 28million additional deaths which is projected to exceed deaths from communicable, maternal, perinatal and nutritional diseases combined by 2030^24^.

### Impact of Covid-19 On Combatting NCDs

The emergence of COVID-19 has triggered an unfamiliar response to a new global threat. Although governments have responded by implementing several social and public health measures to contain the spread of the disease, the preventive health measures, the nature and duration of the outbreak may have direct and indirect effect on non-communicable health outcome^25^. The rapid spread of the disease across many countries has impaired delivery of essential medical services including treatment of hypertension, diabetes, cardiovascular diseases and rehabilitation services^26^. According to the WHO Africa office, Africans with NCDs who become infected with COVID-19 have higher chances of progressing to severe form of COVID-19 and eventually die. This has brought to focus the fact that vulnerable groups are not only elderly but also people with comorbidities. While COVID-19 case fatality rate is quite low (3% as of September 26, 2020^26^, it is important to note that significant proportion of people who died from COVID-19 have underlying non-communicable disease condition^27^.

Various prevention strategies placed in order to mitigate the spread of the novel Coronavirus have had direct and indirect impacts on combating NCDs^28^. One of such impacts is the possible effect on the disruption of regular screening. Combating NCDs in patients requires consistent monitoring and frequent screenings. In patients with Hypertension, checking the Blood pressure regularly is essential in ascertaining the efficacy of anti-hypertensive therapy and predicting the need for continuity of medication or addition of extra medication 29. Patients with Diabetes need to check their Blood sugar regularly but this as well has been negatively affected by COVID-19. Movement restrictions inhibit accessibility to screening centers, clinics and Pharmacies 28.

Although Africa largely depends on the supply of pharmaceutical raw materials from China and India 30, the emergence of COVID-19 has posed significant threat on the production and supply of essential medicines. The preventive measures implemented have disrupted the logistics of pharmaceutical products resulting in limited access to essential medicines including drug use in the management of NCDs. This situation may worsen the health of patients with NCDs and potentially contribute to higher risk of mortality in patients with COVID-19, thus increasing the burden of diseases 31.

COVID-19 has imposed psychological consequences on the mental health of the general population. The resulting depression may have adverse effect on the management of diabetes and other chronic illnesses 32. Studies have revealed that depression is associated with poorer health outcomes in the management of diabetes, hypertension and cancer; patients with diabetes or hypertension or cancer are at increased risk of developing complications and death due to depression 32-34. There is a myriad of conditions that may be associated with depression, thus the psychological impact of COVID-19 prevention strategies on NCDs should not be underestimated.

Social and public health measures implemented may have impact on adoption of unhealthy habits such as tobacco smoking, unhealthy diets (increased consumption of ultra-processed foods), and reduced physical activity 35. Physical inactivity increases the risk of a number of diseases such as Diabetes, hypertension, cancer, obesity, etc. 36. The necessity of regular physical activity cannot be over-emphasized both for young people and adults. Physical activity helps improve metabolic health, brain health, cardiovascular health and overall body function 37. Physical activity may also have a positive effect on the progression of Parkinson's disease^38^. Due to restriction of movement, physical activity may be reduced, affecting the progress against NCDs.

Globally, treatment and diagnostic services for NCDs were commonly disrupted in various countries (about 75% of countries)^30^. The movement restrictions and fear of accessing health facilities has limited the ability of patients to visit their physicians for consultation and treatment. Patients with mental health disorders who may usually require regular visits for evaluation and consultation with their healthcare provider may find it difficult as well, especially in this pandemic when they are vulnerable^39^

COVID-19 prevention strategies in various countries have indeed impacted negatively on NCDs either via access to preventive measures, treatments, prescriptions, etc. [30] and health systems may need to prepare for the aftermath of the COVID-19 pandemic.

### Covid-19, Past Pandemics and NCDs

A plethora of pandemics have swept through time, ranging from black death, the third cholera pandemic, the sixth cholera pandemic, the Spanish flu pandemic, HIV/ AIDS pandemic, Swine flu, Ebola and now COVID-19. COVID-19 has become a frequent topic of discussion across the globe as the number of active cases rise; some leading to death and others to startling recoveries. Each of these pandemics has held mankind for ransom and created an uproar as the presence of vaccines have been unavailable at the time of its outbreak 40.

Judging by the number of deaths caused so far alone, COVID-19 can be compared to pandemics such as the Asian flu which killed an estimated l.lmillion people, and Hong Kong flu pandemic which killed almost 1million people 41. The World has suffered even more severe pandemics in the course of human history. The Spanish flu also called the 1918 flu pandemic was one of the deadliest pandemics in human history, it affected 500million people (almost a third of the world's population at the time) and the number of deaths was estimated to be between 17million and 50million or even possibly up to 100million^42^. The Black Death also called the plague was known as the most fatal pandemic in recorded history and it was known to have killed an estimated 75-200 million people in the 14th century 43.

A study from severe acute respiratory syndrome (SARS) and H1N1 pandemic flu outbreaks 44 revealed substantial rate of psychological distress, post-traumatic stress among health care workers resulting from increased adverse opinion of negative health impact of a new and emerging disease, feeling of isolation and fear of friends and family getting infected 45. Considerable impact of past pandemics on mental health of the general population has also been reported^46^, ^47^ including psychological consequences and depression. Underlying mental health, pregnancy, disability, low income and aging have been identified as factors that contributes to increase susceptibility to poor mental outcomes^44^. The world is currently battling a global pandemic of SARS-CoV-2; the novel coronavirus that causes COVID-19. The pandemic is distinct from the 1918 H1N1 influenza pandemic in that it directly affects (but not limited to) the elderly and those with pre-existing health conditions.

Non-communicable diseases (NCDs) are a group of conditions that includes cardiovascular disease, cancer, diabetes, chronic respiratory diseases and mental disorders. Over the past three decades, death toll from NCDs has increased from 27 million to 38 million and currently makes up 70% of all global mortality (Institute of Health Metrics and Evaluation). NCDs once predominates high income countries, but are now the leading cause of death in both developed and developing countries. 80% of these deaths occur often in low- and middle-income countries (LMICs) where over 70% of world's poorest people live and where access to quality and universal health care services are limited 48. According to the World Bank, 80% of 54 African countries are classified as LMICs. Social, political and economic trends (including national economic activity, population aging, urbanization, globalization and availability of unhealthy products) have shown to be significant contributing agents to increasing NCDs over the last few years 49. These agents work by promoting exposure to ultra processed foods and drinks, alcohol, tobacco products and varying social, economic factors that limits physical activities 50. Studies have shown that NCDs are not entirely self-imposed as it can be passed from one person to another through viral transmission for example in liver or cervical cancer (Lowy and Schiller, 2012) or through built social, economic and environmental conditions and genetic transmission 51-54. While NCDs do not meet the benchmark for infectious disease classification, these associations have led to the admission of the existence of NCD epidemic (CDC, 2016). Trends in past infectious disease outbreaks have revealed a direct or indirect impact on NCD progression through effect on health, social and economic climate 55. The results of these impacts include effect on mental health, health behaviour and lifestyle among health care workers and general population 50, 56. It was reported that hypertension, diabetes, and chronic respiratory problems were the most common comorbidities observed in covid19 patients admitted to critical care units in most countries 56.

### NCDs target and Africa

It is expected that Africa will experience the largest increase in NCD related mortality globally: about 46% of all fatality in Africa is expected to be attributed to NCDs by 2030 57, 58. Unless urgent measures are taken, the rising NCDs burden will add great pressure to the already overstretched health systems and pose a major challenge to the healthcare sector in Africa. The World Health Organization (WHO) in April 2011 published the WHO Global status report on NCDs 2010 which provided a description of the global burden of NCDs, their risk factors and determinants 59. This report led to its adoption at the first United Nations (UN) General Assembly high level meeting on NCDs in 2011 59, 60. Consequently, the NCDs country profiles were published in September 2011 to provide individual member countries NCD situational analysis 60. This report ultimately resulted in the NCDs Global Action Plan 2013–2020 which was released in 2013 and further reinforced by the 2030 agenda 59.

Furthermore, In July 2014, the NCD country profiles was published to provide an update of each member state NCD status so as to assess progress towards the NCDs Global Action Plan 2013–2020 and identify blockages and priority actions 58. Ten (10) indicators made up of 18 targets were then developed by the WHO to promote monitoring and evaluation by each member states in their respective NCDs prevention and control progress 59, 60.

These indicators and targets would also form the basis for WHO reporting at the 2017 UN General Assembly and at the 2018 high level UN General Assembly meeting. In addition, another NCD country profiles report was published in September 2015 which aimed to figure out individual member countries progress towards the NCDs targets and indicators 61.

From the report, it was evident that globally, 14 countries had not achieved any indicator, 12 of which were in Africa while 20 countries had only achieved one indicator, 15 of which were in Africa 62. African countries are already disoriented in achieving their NCDs targets and since the inception of Covid-19 pandemic, primary healthcare settings and health systems have been overburdened and this can of course militate against effective treatment of NCDs and without immediate strategies and interventions, African countries may not be able to achieve the SDG 3.4 target of reducing by one-third, premature mortality from chronic non-communicable diseases by the year 2030.

## Conclusion

Health professionals should be adequately provided with PPEs to improve their protection against COVID-19 infections. This would give patients with NCDs the confidence of going for check-ups knowing they have minimal risk of exposure to the virus. More health professionals should be employed to continue the provision of care for patients with COVID-19 while the rest focus on effectively responding to NCDs. In countries where employment and recruitment of more health professionals becomes challenging due to financial constraints, alternative measures like the use of telemedicine for self-management and the follow-up of patients with NCDs could be implemented 63.

Health systems and infrastructures in African countries should be expanded and equipped with donor support to accommodate COVD-19 and NCDs patients at the same time. Also, medication delivery services and drones could be used to deliver NCDs medications and urgently required treatments when necessary. There should be increased community COVID-19 response teams of all African countries greatly hit by the pandemic to enhance community support for persons living with NCDs and to reduce social stigmatization. Achieving the NCD targets in Africa requires population-based interventions by not only the government, but also a collaborative effort with private institutions in encouraging healthier patterns, lifestyle and daily habits. Achieving economic development in a nation depends greatly on the health of members of the population; hence, achieving these NCDs target should form the core of National Strategic plans of various countries.

## Figures and Tables

**Figure 1:**
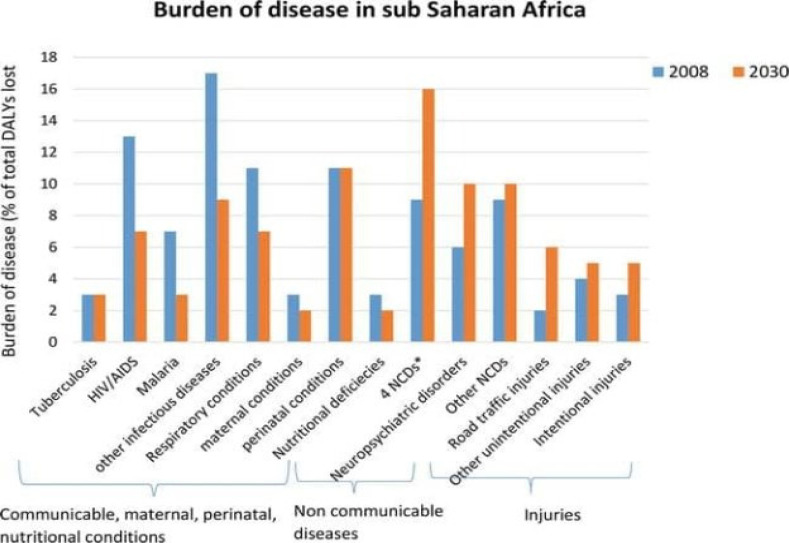
Projected Burden of diseases in Sub-Saharan Africa, 2008 and 2030 (Source of data: Authors from WH, 2008)

**Figure 2:**
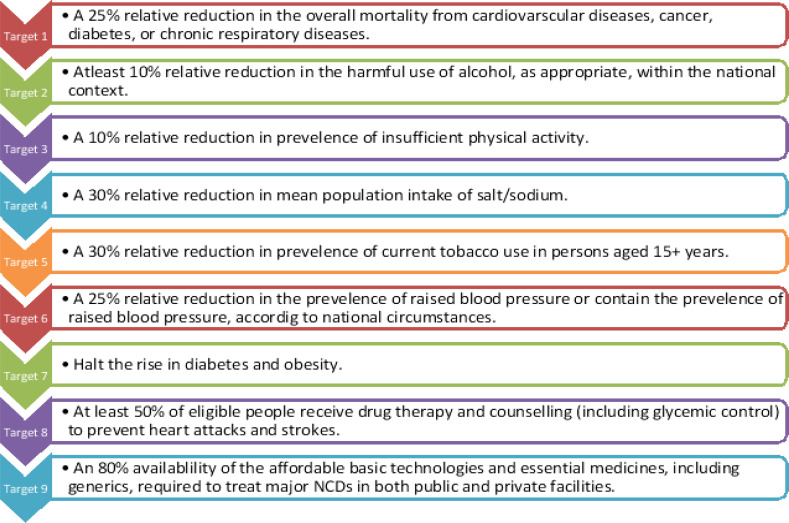
Non-communicable Disease (NCD) Targets (Adopted from: WHO Non-communicable diseases: Campaign for action- meeting the NCD targets)

**Table 1:** Past and Current Pandemics

Name of Pandemic/Period	Cause of transmissions/deaths	Countries	Further information/Measures to follow.
Antonine plague (165 AD)	Unknown/5 million	Anatolia, Egypt, Greece, Italy	Believed to be outbreak of smallpox, appearance of boils sounds like smallpox/no treatment
Plague of Justinian 541-542 AD	Bubonic plague/25 million	Constantinople, Eastern Mediterranean	Flea/insect control/sanitation of ports / ships Overall personal hygiene; bacterial infection Yersinia pestis transmitted through fleas
Black Death 1346-1353	Bubonic plague/75-200 million	Asia, Africa, Europe	Carried by fleas Recurrence until twentieth century
Third cholera pandemic 1852-1860	Cholera/contaminated water/1 million	India, Asia, Europe, North America, Africa	Vibrio cholerae contaminated water Treatment of water/daily monitoring of water quality parameters
Flu pandemic 18891890	Influenza A virus/H2N2/H3N3/1 million	Central Asia, Canada, Greenland	Bed rest; ample fluids; nourishing food; treatment through alcohol to quinine, salicylates
Sixth cholera pandemic 1910-1911	Cholera/800,0	Middle East, North Africa, Eastern Europe, Russia	Transmitted through water contaminated with faeces and food; disinfection of water; separating water supply lines from human sewage; care for infants
Flu pandemic/Spanish flu 1918-1919	Influenza/20-50 million	Europe, Australia, Africa, North America	Social distancing; wearing masks; avoid public gatherings; care for health care workers, nurses, doctors, etc.; community spread measures; quarantine; isolation; economy
Asian flu 1956-1958	Influenza/H2N2/2 million	China, Singapore, Hong Kong, USA	Infections in children from schools; deadly to pregnant women and elderly with existing lung and heart diseases; economy
Flu pandemic/Hong Kong flu 1968	Influenza/H2N2/1 million	Hong Kong, Singapore, Vietnam/Philippines, India, Australia, Europe, USA	Human-to-human transmission Social distancing; isolation; treatment with ample fluids; nutritional food; economy
HIV/AIDS pandemic 2005-2012	HIV/AIDS/36 million	Africa, globally 131 countries	Social distancing; no heterogeneous sex; use of condoms; personal hygiene; challenge of bringing awareness; still existing; economic pressure on developing countries
COVID-19 pandemic 2019-till date	613,213 as of 21/07/2020	China, Europe, USA, South America, Africa, Gulf countries, Russia	Characteristics of virus are changing Fast-spreading; symptoms appear after 6 to 7 days Preparedness; decision-making Social distancing; masks; separate elderly from children No public gatherings Learn to live with it with full precautions
